# Supplementary material for: Foliar Application of Selenium Associated with a Multi-Nutrient Fertilizer in Soybean: Yield, Grain Quality, and Critical Se Threshold
Source: Plants (Basel). 2023 May 18;12(10):2028. doi: 10.3390/plants12102028 (PMC10221896; doi:10.3390/plants12102028)
Supplement: Supplementary file 1 [file plants-12-02028-s001.zip › plants-2344161-supplementary.pdf]

Supplementary Material

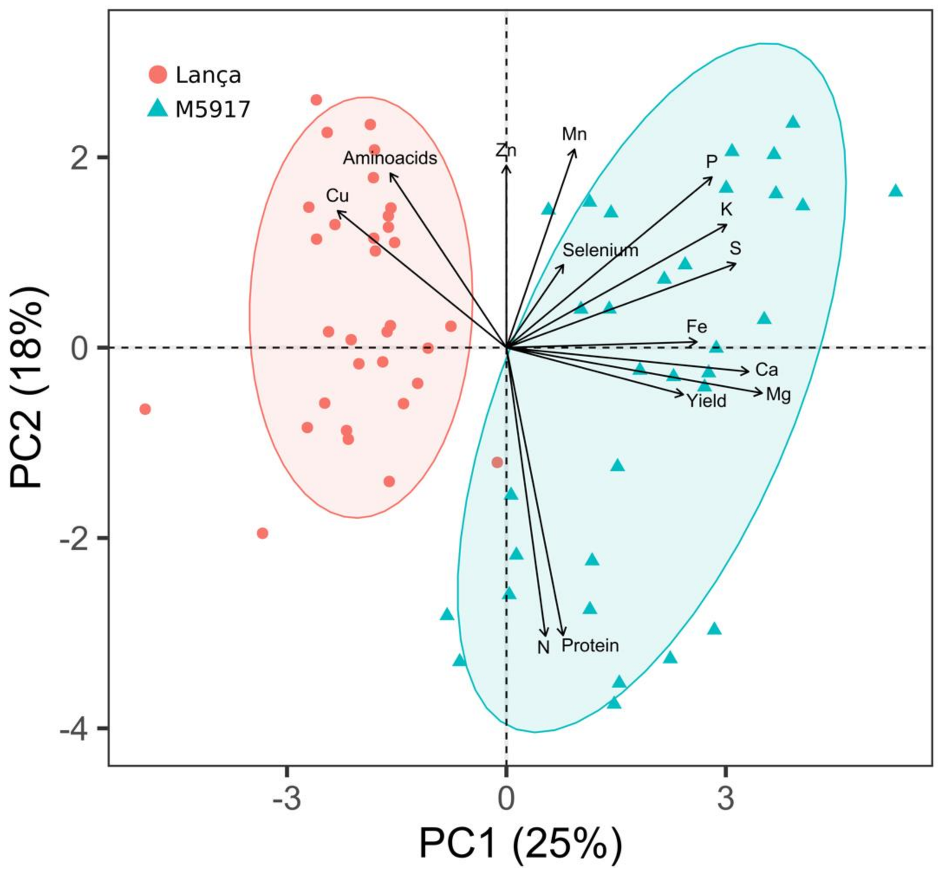

Figure S1. Biplot of principal component analysis (PCA).

Table S1. Summary of F Test (ANOVA) and regression MNF for macronutrient, micronutrient, protein, and amino acid analyses.

Variable	Significance			Adjusted Equation/Mean	R^2 (%)	CV (%)
	Rate	MNF	Rate*MNF			
**58I60 LANÇA**						
N	ns	ns	ns	ns	-	2.2
P	*	ns	*	With MNF: no adjusted<br>Without MNF: y = 5.4726 - 0.0029x	-<br>44	2.2
K	ns	ns	*	no adjusted	-	2.4
Ca	ns	ns	ns	ns	-	5.8
Mg	ns	ns	ns	ns	-	3.4
S	ns	ns	***	With MNF: y = 2.4497 + 0.0012x<br>Without MNF: y = 2.5319 - 0018x	89<br>68	1.9
Cu	ns	ns	ns	ns	-	7.9
Fe	ns	ns	ns	ns	-	4.2
Mn	ns	ns	ns	ns	-	3.6
Zn	ns	*	ns	ns	-	5.9
Protein	ns	ns	ns	ns	-	2.2
Amino acids	*	ns	*	With MNF: y = 139.1267 – 0.3811x<br>Without MNF = no adjusted	60<br>-	7.6
**M5917**						
N	*	ns	ns	General equation: $y = 69.1895 - 0.1130x + 0.0011x^2$	-	2.2
P	*	*	*	With MNF: $y = 5.4893 + 0.0193x - 0.0002x^2$<br>Without MNF: no adjusted	50<br>-	3.0

K	*	ns	ns	General equation: $y = 17.5397 + 0.0459x - 0.0005x^2$	77	2.9
Ca	ns	ns	ns	ns	-	6.7
Mg	ns	ns	ns	ns	-	4.6
S	*	*	ns	General equation: $y = 2.5731 + 0.0074x - 0.0001x^2$	63	4.2
Cu	ns	*	*	With MNF: $y = 10.1170 + 0.0387x - 0.0007x^2$	80	
				Without MNF: no adjusted	-	
Fe	ns	ns	ns	ns	-	6.1
Mn	*	ns	ns	General equation: $y = 28.0131 + 0.0190x$	67	3.4
Zn	*	ns	*	General equation: $y = 39.7419 + 0.1295x - 0.0013x^2$	55	3.1
Protein	*	ns	ns	General equation: $y = 43.3434 – 0.0707x + 0.0007x^2$	94	2.2
Amino acids	*	ns	*	no adjusted	-	6.8

* Significance 0.05; ns = no significance by F test; CV = Coefficient of variation; R^2 = regression coefficient
